# Factors Influencing Exercise Engagement When Using Activity Trackers: Nonrandomized Pilot Study

**DOI:** 10.2196/11603

**Published:** 2019-10-24

**Authors:** Amanda Jayne Centi, Mursal Atif, Sara Bersche Golas, Ramin Mohammadi, Sagar Kamarthi, Stephen Agboola, Joseph C Kvedar, Kamal Jethwani

**Affiliations:** 1 Pivot Labs Partners Healthcare Boston, MA United States; 2 Department of Mechanical and Industrial Engineering Northeastern University Boston, MA United States; 3 Department of Dermatology Massachusetts General Hospital Boston, MA United States; 4 Harvard Medical School Harvard University Boston, MA United States

**Keywords:** activity trackers, exercise, engagement

## Abstract

**Background:**

It is well reported that tracking physical activity can lead to sustained exercise routines, which can decrease disease risk. However, most stop using trackers within a couple months of initial use. The reasons people stop using activity trackers can be varied and personal. Understanding the reasons for discontinued use could lead to greater acceptance of tracking and more regular exercise engagement.

**Objective:**

The aim of this study was to determine the individualistic reasons for nonengagement with activity trackers.

**Methods:**

Overweight and obese participants (n=30) were enrolled and allowed to choose an activity tracker of their choice to use for 9 weeks. Questionnaires were administered at the beginning and end of the study to collect data on their technology use, as well as social, physiological, and psychological attributes that may influence tracker use. Closeout interviews were also conducted to further identify individual influencers and attributes. In addition, daily steps were collected from the activity tracker.

**Results:**

The results of the study indicate that participants typically valued the knowledge of their activity level the activity tracker provided, but it was not a sufficient motivator to overcome personal barriers to maintain or increase exercise engagement. Participants identified as extrinsically motivated were more influenced by wearing an activity tracker than those who were intrinsically motivated. During the study, participants who reported either owning multiple technology devices or knowing someone who used multiple devices were more likely to remain engaged with their activity tracker.

**Conclusions:**

This study lays the foundation for developing a smart app that could promote individual engagement with activity trackers.

## Introduction

### Background

Despite the well-recognized benefits of physical activity, millions of people are physically inactive, and the prevalence of physical inactivity is increasing, placing people at a greater risk for obesity and many other cardio metabolic disorders [1,2]. In 2016, physical inactivity was reported as the fourth leading cause of mortality [3]. Yet, as we age, we spend more time physically inactive. It is estimated that Americans aged 20 to 29 years spend 55% of their time inactive, whereas those aged 70 to 79 years spend 67% of their time inactive [4]. Currently, there is much research into methods to not only increase physical activity but also forge sustainable physical activity patterns. Technology plays an important and promising role in personal activity tracking. Wearable activity trackers have been championed as powerful personalized health management tools because of their “low cost, wide reach, and apparent effectiveness,” and the commercial market for such devices is large and expanding [5,6]. In 2013, 1 out of every 5 US adults surveyed reported using “some form of technology to track their health data,” including medical devices, mobile phone apps, or Web-based tools [7]. Individual consumers and health care providers recognize the potential benefits of wearable activity trackers, which may be used to monitor many health indicators, including diet, physical activity, and sleep [8]. In addition, activity trackers can be beneficial in aiding with chronic disease management by promoting behavioral health changes encouraged by health care providers, such as increasing physical activity [5]. It has been reported that the use of technology to monitor physical activity was associated with higher levels of activity [8]. However, the potential benefits derived from the use of activity trackers are challenged by the limited and transient adoption of these devices, which requires sustained use to achieve their intended effect [8]. Although it has been well documented that most users lose interest in using trackers not long after purchase, specific reasons for this remain to be deciphered [9]. Currently, little is known about the factors associated with the adoption and sustained use of activity trackers or the barriers that limit the effectiveness of these devices in people’s efforts to increase physical activity and improve health. Perceived barriers to physical activity have been previously studied, and it has been demonstrated that motivational factors are associated with physical activity level [1]. Lack of time, fatigue, and a dislike for exercise are some of the barriers that reflect a lack of motivation to engage in physical activity [1]. If the characteristics and patterns that lead to a person becoming disengaged with his or her tracker are also better understood, interventions may be created to maintain engagement. However, little is known about when a person may disengage with his or her tracker and what personal characteristics may be influencing that decision.

### Objective

Therefore, purpose of this study was to understand reasons for engagement and disengagement associated with the use of activity trackers in an overweight population. We chose an overweight population as our first use case, as the benefits of maintaining or even increasing physical activity in this population have been well established, whereas sustaining interventions are lacking [10-12].

## Methods

### Study Design

This was a 9-week, nonrandomized pilot study designed to explore activity tracker engagement patterns to guide and inform future work into the development of a predictive algorithm to facilitate user engagement with trackers.

### Study Population and Setting

Participants (n=30) aged 18 years and older, with a body mass index of 25 kg/m^2^ or greater, were recruited from a Massachusetts General Hospital clinic. After screening into and consenting to the study, participants were directed to the study website (wellocracystudy.org), where they were asked to read through information regarding the study and types of activity trackers available to use for the 9-week study and keep after study completion. Participants could choose from the following FitBit activity trackers: Charge, Flex, One, or Zip. After choosing a device, study staff assisted with setup as necessary.

### Data Collection

Participants were asked to wear the activity tracker continuously during the 9-week study period. The first week was used as a run-in period to determine the participant’s baseline average daily steps. A step goal was then set at 10% above this average. Each participant’s step goal was unique to the participant on the basis of the participant’s activity during week 1. Participants were sent a short message service text message to change their step goal to this amount for the remaining 8 weeks. During the 8 weeks, minimal contact was made by study staff with participants so that their habits of using the activity tracker could be assessed. Study staff would download the data weekly from the FitBit application programming interface. If it was found that no data were being collected from the activity tracker, study staff reached out to the participant and attempted to provide support on pairing the device and uploading data in the FitBit app. If a participant indicated not using the device, reasons for nonuse and engagement were documented and explored in depth with a trained staff member. After the study, participants completed a closeout survey either on the Web or in paper format and underwent a phone interview with a neuropsychologist to gather information about their experiences during the study, which was transcribed for analysis. Baseline and closeout questionnaires included questions about their thoughts about and perceived barriers to exercise and activity—Behavioral Regulation in Exercise Questionnaire (BREQ-2) [13] and Barriers to Being Active (BBA) [14]), Prochaska’s Stage of Change [15], and general health questions (Patient-Reported Outcomes Measurement Information System, PROMIS Global-10) [16]. As episodes of severe depression can impact physical activity in ways beyond the scope of this intervention, all participants underwent a screening—Patient Health Questionnaire (PHQ-8) [17]. In addition, technology use, ownership, and demographic data were collected at baseline only. The BREQ-2 is designed to gauge the extent to which people’s reasons for exercise are internalized and self-determined on the basis of the following categories: amotivation, external, introjected, identified, intrinsic. The BBA assesses whether participants gauge certain categories as reasons for inactivity, including energy, willpower, time, and resources. For each category, a score of 5 or greater would indicate that category as a substantial barrier to a person’s ability to exercise. The PROMIS Global-10 is a 10-item survey that seeks to assess health care–related quality of life along 2 metrics, physical and mental health for all participants, and it was administered at enrollment and closeout. The PHQ-8 is a brief survey of a person’s depression status, whereas Prochaska’s Stage of Change assesses a person’s state for changing current habits and behaviors. In addition to the survey questions, participants were asked to complete a poststudy phone interview with trained staff. Although the questions were open ended in general, the main purpose of these interviews was to decipher more specific reasons for how participants engaged with their activity tracker during the study period. These interviews were recorded and transcribed. Each interview lasted between 30 and 45 min in length.

### Data Analysis

For analysis, participants were divided into 3 *a posteriori* “engagement groups” on the basis of the percent of days they met their step goal. Step data were collected and analyzed to determine average number of steps, amount of time participants wore their device, and the percent of days a person met his or her step goal. Each participant’s step goal was determined by taking the participant’s average number of steps from Week 1 and adding 10% to this number. A weekly average of steps per day was then calculated for each participant. Questionnaires were scored according to their standard practice. In instances where 1 of the questionnaires was skipped, values from the enrollment questionnaire were carried over for analysis purposes. These instances are indicated in the descriptions below. Qualitative data from closeout interviews were analyzed by trained staff in qualitative analysis who conducted a thematic analysis of the transcribed interviews for key patterns to the motives and barriers to using activity trackers. Means and SDs were used for continuous variables. Categorical variable percentages were calculated as percent of group total.

## Results

At the beginning of the study, a total of 30 participants were enrolled. Among them, 21 participants completed both enrollment and closeout procedures as part of the study. The remaining 9 participants were lost to follow-up. Baseline characteristics of all enrolled participants (n=30) are summarized in Table 1.

Overall, the participant distribution comprised 60% female, 70% white, 47% employed, and 60% individuals with at least some education post-high school. As the distribution of engagement groups was skewed—Shapiro–Wilk *P* value≤.01; median (Q1, Q3)=37.3% (26.2%, 51.3%); range=0%-83.7%. Groups were defined by quartile: the bottom 2 quartiles= “Low engagement,” the third quartile=“Medium engagement,” and the upper quartile=“High engagement.” Patients lost to follow-up were classified as “Nonengaged,” as they were not engaged enough with the tracker to complete the 9-week study. Nonengaged participants were included in the study analysis to try to determine initial characteristics that may be identified before the study to keep similar future participants engaged during a follow-up study. Though Fisher Exact test revealed a statistical significance among engagement groups for Marital Status (*P*=.01), engagement groups were statistically similar on all other demographic variables. All but 3 participants chose to use the FitBit Charge model. A person in the high and medium groups used the FitBit One, whereas a person in the nonengaged group used the FitBit Zip.

Over the 8 weeks of data collection, the number of participants who met their weekly step goals was low. Overall, less than 50% of participants met their step goal each week (Table 2).

The number of days participants met their step goal over the study period ranged from 3 days to 38 days, with an average of 20.2. As there seemed to be a range in participants meeting their step goal, and we wanted to determine facilitators or barriers to meeting this goal, the Engagement Level categories described above were used to look for patterns and characteristics that could be used to profile how and why participants engaged in certain manners. Of the 30 participants enrolled, 28 completed the enrollment questionnaire, and 21 complete the closeout questionnaire. The patient Enrollment and Closeout Questionnaire results are summarized below.

**Table 1:** Participant demographics (N=30).

Variable		Value
Age (years), mean (SD)		48.96 (9.54)
Gender (male), n (%)		9 (30)
**Body mass index at enrollment, mean (SD)**		32.48 (4.59)
	Range	25-41.2
**Race, n (%)**		
	White	21 (70)
	Nonwhite	9 (30)
**Marital status, n (%)**		
	Married	8 (26.7)
	Divorced/separated	8 (26.7)
	Single (never married)	8 (26.7)
	Living with partner	3 (10)
	Widowed	1 (3.3)
	No response	2 (6.7)
**Education, n (%)**		
	12 years or completed high school or General Education Diploma	5 (16.7)
	Some college	5 (16.7)
	College graduate	9 (30)
	Posthigh school	2 (6.7)
	Postgraduate	2 (6.7)
	Less than high school	3 (10)
	Unknown	4 (13.3)
**Employment status, n (%)**		
	Employed/self employed	15 (50)
	Disabled	5 (16.7)
	Unemployed	5 (16.7)
	Student	1 (3.3)
	Retired	1 (3.3)
	Unknown	3 (10)

**Table 2:** Percent of participants meeting their step goal (based on week 1 data), by week, over the course of the study.

Week	Patients who met goal (%)
2	23
3	50
4	45
5	23
6	41
7	32
8	23
9	27

### Friends and Family Who Track

During enrollment, participants were asked if they knew anyone who used trackers. We found that participants with friends or family who used trackers were more engaged over the course of the study. Though not significant (*P*=.09), less engaged participants were less likely than those who continued engagement to have friends or family members with trackers: 50% to 80% of those who continued in the study have friends, family, or both who track, whereas the same can be said for only 14% of the nonengaged.

### Technology Ownership

**Figure 1:**
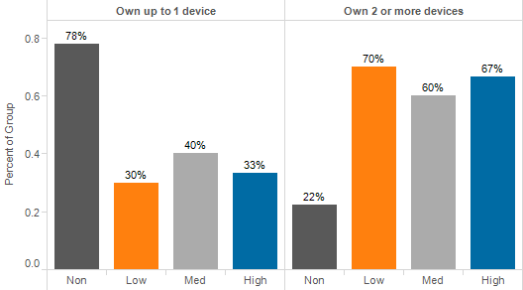
Device ownership, by engagement level.

Participants were asked whether they owned particular items of technology, such as a desktop computer, a laptop computer, or a tablet. Nonengaged participants were more likely to own only 1 device, whereas other engagement levels were more likely to own 2 or more devices (Figure 1, *P*=.18).

### Stage of Change: Enrollment and Closeout

**Figure 2:**
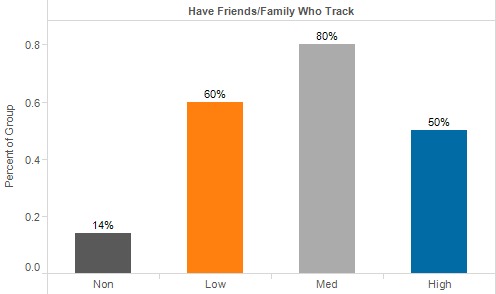
Stage of Change at enrollment.

**Figure 3:**
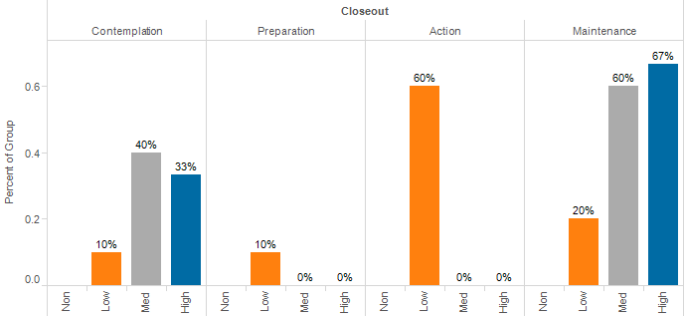
Stage of Change at closeout, by engagement level.

At enrollment, there were no differences in Stage of Change observed between groups (Figure 2, *P*=.67). At closeout (see Figure 3), there was a significant difference in Stage of Change between the groups (*P*=.04).

### Patient Health Questionnaire-8: Enrollment and Closeout

There was no difference in PHQ-8 scores at enrollment (non=8±5.7, low=4.5±3.6, medium=5.2±5, and high=8.2±8) or closeout (low=6±5.3, medium=6.4±2.9, and high 6±6.4). Though not significant, the High-Engagement group saw a decrease in PHQ score (*P*=.36), whereas the low- and medium-Engagement groups both saw a slight increase (*P*=.39 and *P*=.50, respectively). Total scores of 10 or greater are indicative of depression, whereas scores of 20 or greater are indicative of severe depression. At enrollment, there were 5 participants who met the score for depression (non=2, low=1, medium=1, and high=2), whereas at closeout, there were 4 participants who did not (low=2, medium=1, high=1).

### Behavioral Regulation in Exercise Questionnaire

Table 3 presents the percentage of each group who had each degree of self-determination as its highest BREQ category score at Enrollment and Closeout. Although most patients had “identified” or “intrinsic” as their highest score, no group had “amotivation” or “external” as its highest score.

Overall, the averages for less self-determined categories (Amotivation, External) increased, whereas the averages for more self-determined categories (Identified, Intrinsic) decreased from enrollment to closeout.

**Table 3:** Highest Behavioral Regulation in Exercise Questionnaire-2 Category score by group at enrollment and closeout (percentage of group).

Group	Enrollment classification				Closeout classification			
	Tied, %	Introjection, %	Identified, %	Intrinsic, %	Tied, %	Introjection, %	Identified, %	Intrinsic, %
Non	—^a^	14	29	57	—	—	—	—
Low	20	10	40	30	10	—	70	20
Medium	—	—	60	40	—	20	40	40
High	17	—	33	50	17	17	50	17

^a^Not applicable.

### Barriers to Being Active

**Figure 4:**
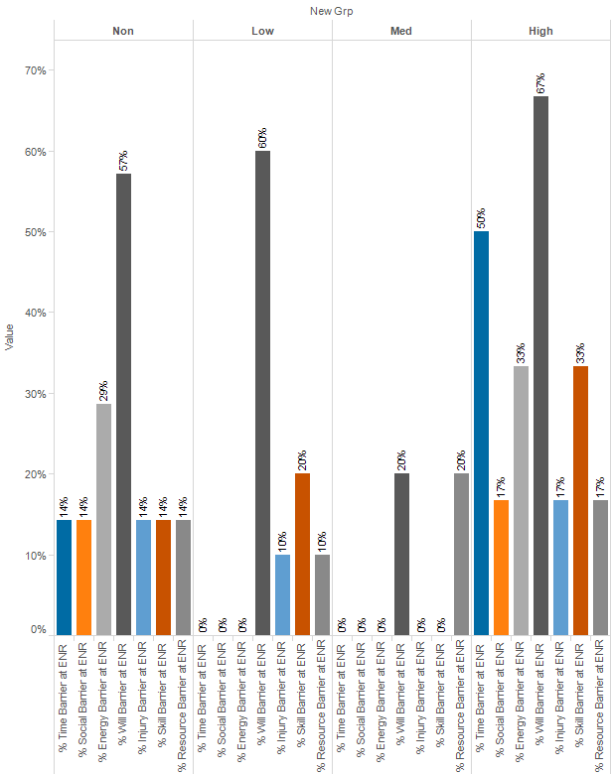
Percent of group for whom category is a barrier, by engagement level at enrollment. ENR: enrollment; Grp: group.

**Figure 5:**
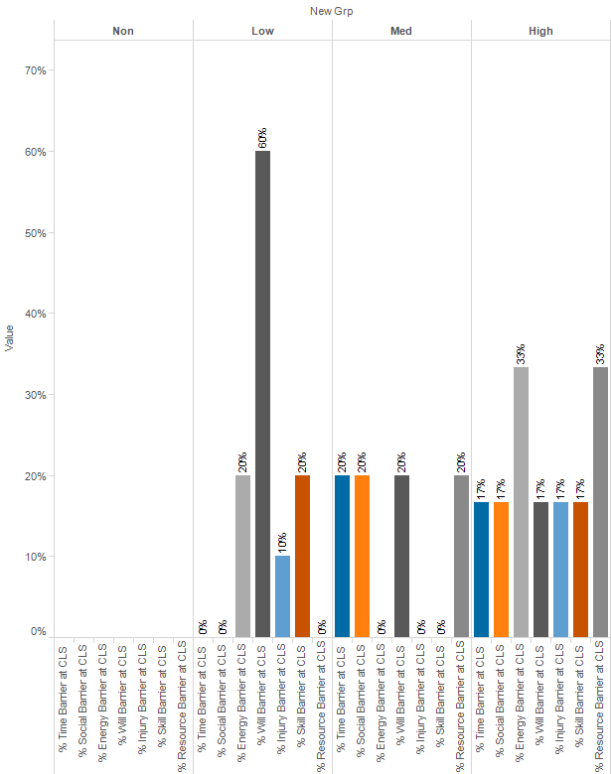
Percent of group for whom category is a barrier, by engagement level at closeout. CLS: closeout. Grp: group.

At enrollment, lack of willpower was the highest average category score for all 4 engagement groups, as well as the most frequent barrier (Figure 4). At closeout, lack of willpower still had the highest average category for the low- and medium-engagement groups, whereas the high-engagement group showed a decrease in this score, with lack of resources having the highest average. A similar pattern is seen with regards to percentage of each group for whom lack of willpower is a barrier (Figure 5).

Between groups at enrollment (ENR), lack of time was the only significantly different category among engagement groups (Table 4; *P*=.03). However, this difference did not remain at closeout (CLS). Within groups, there were no significant differences for any group or category, and most remained similar for each category, with the exception of lack of energy for the low engagement group and lack of time and willpower for the high-engagement group. Between and within groups *P* values are presented in Table 5 below.

**Table 4:** *P* values for between (B) and within (W) group differences, by group, category, and time point (enrollment vs closeout).

Category	Enrollment (B)	Closeout (B)	Low (W)	Medium (W)	High (W)
Lack of time	*.03^a^*	.26	1.00	1.00	*.55*
Social barriers	.40	.26	1.00	1.00	1.00
Lack of energy	.10	.53	*.47*	1.00	1.00
Lack of willpower	.47	.18	1.00	1.00	*.24*
Fear of injury	1.00	1.00	1.00	1.00	1.00
Lack of skill	.68	.77	1.00	1.00	1.00
Lack of resources	1.00	.12	1.00	1.00	1.00

^a^Italics indicate significance.

**Table 5:** *P* values for between (B) and within (W) group differences, by group, category, and time point (enrollment vs closeout): category scores. Italics denote significance.

Category	Enrollment (B)	Closeout (B)	Low (W)	Medium (W)	High (W)
Lack of time	.43^a^	.96	.40^b^	.17	.18^c^
Social barriers	.19	.92	.66	.21	1.00
Lack of energy	.47^d^	.86	.29	.14	.62
Lack of willpower	.76	.59	.83	.70	.20
Fear of injury	.51	.40	.79	1.00	.71
Lack of skill	.36	.48	1.00	1.00	.59
Lack of resources	.13	*.03*	.52	.07	1.00

^a^Kruskal-Wallis test.

^b^Mann Whitney *U* test.

^c^Paired *t* test.

^d^One-way analysis of variance.

For category scores, between and within group comparisons (Table 5) show the groups to be similar across categories and timepoints, with the exception of between groups scores for lack of resources at closeout.

### General Perceived Health

Scores from the PROMIS Global-10 are reported in Table 6. Scores are presented for Physical (Global Physical Health, GPH) and Mental (Global Mental Health) health scores. The participants in the high-engagement group were the only participants in whom an increase in GPH was observed from enrollment to closeout (*P*=.04)

Closeout interviews were conducted on 15 participants. From these interviews, common themes that helped and hindered tracker use were compiled. Common influencers to high or low tracker use included participant’s health status, pain level, weather, emotional state, and daily agenda/routine. Other barriers to use included a preference for more sedentary activities, insufficient space to exercise, and difficulty starting a new routine. Motivators that helped participants increase their activity were categorized as either extrinsic or intrinsic. Extrinsic motivators included personal/internal goals (lose weight, improve health and energy level) and externally imposed goals (tracker goal and rewards, social comparison), whereas the largest intrinsic motivator reported was an enjoyment of being active. From the closeout interviews, 4 criteria were identified to more likely trigger a change in activity level and routine. These included the following: (1) a routine that allowed for exercise without significant barriers, (2) an extrinsic motivator to pursue an activity, (3) a clear and specific personal activity goal, and (4) an extrinsic accountability mechanism (personal trainer, workout buddy, and activity tracker).

**Table 6:** Global Physical Health/Global Mental Health Scores at enrollment (n=27) and closeout (n=20), by engagement level. Italics indicate significance.

Category		Engagement level				Between-group *P* value
		Non (n=7)	Low (n=10)	Medium (n=5)	High (n=5)	
**Global Physical Health, mean (SD)**						
	Enrollment	36.2 (8.05)	41.8 (7.61)	36.9 (8.92)	37.5 (8.33)	.49
	Closeout	—^a^	42.1 (6.88)	43.7 (7.83)	47.0 (6.69)	.45
Within-group *P* value		—	.89	.15	*.04*	—
**Global Mental Health, mean (SD)**						
	Enrollment	43.5 (5.22)	45.0 (5.12)	51.3 (3.77)	42.0 (7.76)	.06
	Closeout	—	43.0 (4.77)	48.3 (2.50)	45.9 (5.13)	.11
Within-group *P* value		—	.15	.18	.29	—

^a^Not applicable.

## Discussion

To our knowledge, this is one of the first studies to take an in-depth look at the reasons participants choose to engage with activity trackers and work to develop attributes that can be identified to better predict a person’s engagement. In general, we were able to determine that those who reported owning more devices were more likely to be engaged with their activity tracker than those with less technology ownership. In addition, those who reported knowing someone who used a tracker were also more likely to remain engaged with their activity tracker. Both of these findings would point to a need for participants to feel a level of comfort with either using the device or having support to help them with the device as a method of increasing engagement. The findings may also point to a need for very simple activity trackers to be able to reach a wider range of people. If there is a low technology barrier for a person to overcome, the person may not need as much experience or support to sustain use. Ease of use was a reason for choosing the FitBit platform for this study, and it has been noted in other studies as well [18]. These reasons can be added to previous reports, which indicate that former users of activity trackers indicated learning all they could, and former users specified trackers not helping them achieve goals as reasons for disengaging from their tracker [19]. A common theme we heard from participants at closeout was a benefit of receiving an actual measure of their physical activity. This is similar to what has been observed in previous reports. In a study on acceptance of commercially available wearable activity trackers among adults over 50, participants reported that the most beneficial aspect of the using an activity tracker was increase in self-awareness of activity levels [3]. In addition, a recently published study noted that over 81% of people using trackers thought it made them more physically active [19]. If we are able to manipulate this self-awareness, it may be another useful tool for keeping participants engaged, at least for a certain amount of time. However, from this study and previous studies, there also seems to be a need for continuation of learning to sustain use [19,20]. However, methods of how this information can be used still need investigation. In addition, it is important to balance the self-awareness of activity with perceived barriers that the participant may experience. Although using the activity tracker helped keep the goal of increasing activity at the top of participants’ minds, but for most, the tracker was not a sufficient motivator to overcome personal barriers and achieve a significant increase in activity (change in routine). During closeout interviews, we were able to separate participants by those who seemed to be motivated by extrinsic versus intrinsic motivators. We found that those who expressed an extrinsic motivator to pursue an activity were more influenced by wearing their activity tracker, whereas those intrinsically motivated to pursue an activity were less likely to express being affected by tracker goals. These participants showed more commitment to making changes to their routine and increasing their activity level, albeit small changes. The fourth poignant point was if the participant did not already have another trigger of activity, such as working with a trainer or other personal fitness/activity goal that worked for the participant, the tracker was more likely to trigger a change in activity level for the participant. Although not explored during this study, previous research has indicated that if a person is not meeting his or her activity goals, the use of a tracker can be discouraging [21]. It would be interesting to pursue this in a follow-up study to determine if a combination of how people are motivated and the feedback they receive on achieving their goals affects overall engagement with trackers. Although this study was short, 9 of the 30 enrolled participants dropped out before the end of the study period. We hypothesize that as participants were given the activity tracker in the beginning of the study and were permitted to keep the device, participants were not as motivated to remain in the study and complete closeout surveys without further recompense. We have modified further phases of this study to deliver compensation at additional timepoints to attain a more complete data set. As an extension to this study, we plan to develop a machine learning–based app to encourage tracker users to stay engaged with exercise. We will look to include questions to elucidate the type of information that could assist in determining methods for keeping participants engaged dependent on whether they are driven by more intrinsic versus extrinsic motivators. This study had a few limitations. Target enrollment was low, and all participants were recruited from the same lab, which may limit the generalizability of the study. In addition, a larger sample population may have resulted in more significant results. Socioeconomic status (SES) was not collected from participants; however, education, which can affect SES, was used as a proxy with no difference between engagement group and education reported. Finally, participants choosing to be in this study may also be more likely to engage with a FitBit tracker compared with those not choosing to participate in the study. Overall, as part of this study, we were able to gain many insights into why overweight participants may or may not engage with their activity tracker. This information will be used to create an algorithm to better sustain engagement with activity trackers.

## Abbreviations

BBA: Barriers to Being Active
BREQ-2: Behavioral Regulation in Exercise Questionnaire
GPH: global physical health
PHQ: Patient Health Questionnaire
PROMIS: Patient-Reported Outcomes Measurement Information System
SES: socioeconomic status
